# T188. MASSIVE OPEN ONLINE DISCOVERY ‘MOOD’ FOR SCHIZOPHRENIA RESEARCH

**DOI:** 10.1093/schbul/sby016.464

**Published:** 2018-04-01

**Authors:** Madhavi Ganapathiraju, Narayanaswamy Balakrishnan

**Affiliations:** 1 University of Pittsburgh; 2 Indian Institute of Science

## Abstract

**Background:**

We have recently presented Schizophrenia Interactome, i.e., the network of protein-protein interactions (PPIs) of schizophrenia associated genes. PPIs predicted by our High-precision Protein-Protein Interaction Prediction model (HiPPIP) which uses machine learning to classify features of protein-pairs such as colocalization, coexpression, common molecular functions and biological processes could explain the apparent discordance between modern and historical genetic basis of Schizophrenia (published in npj Schizophrenia), and also were instrumental in discovering that OASL interacts DDX58 to activate the RIG-I immunity pathway during viral infection. These novel predicted PPIs were found to be highly accurate based on computational and experimental validations, and gave insights into possible functions of SZ genes that previously had no-known functional information. Even a single novel PPI can have enormous impact on advancement of biology, when translated effectively. How can we ensure that 500+ of these novel PPIs of schizophrenia interactome are translated effectively?

**Methods:**

We developed a platform for Massive Open Online Discovery for Schizophrenia Research, or “MOOD for Schizophrenia Research”, that brings together trained biologists and bioinformaticians including those who are currently not affiliated with research labs (non-research students, PhDs who gave up science careers for administrative/corporate jobs or to take care of families, etc), as well as scientists who are active researchers, to hypothesize, discuss and prioritize the novel PPIs. Hypotheses are written as nanopublications with authorship credit. We are developing a number of features on the portal that allow and encourage scientists to create knowledge around the predicted PPIs and be recognized and given credit.

**Results:**

The first version of our website that disseminates the Schizophrenia Interactome is receiving hundreds of unique users each month. We have since developed MOOD for Schizophrenia Research and will present the key features of the website in this work. Each PPI can be viewed, researched on and written about, by participating scientists. Comprehensive information about the proteins in the PPI regarding their known functions, pathways, diseases and drug associations, is made readily available to scientists, allowing them to hypothesize the importance of the specific PPI. We present methods that we employ to promote collaboration in this work. Initially, the portal starts with a few members and it grows through referral webs (i.e. current members invite new members). The portal has a number of features that recognize and thus entice users to participate. We will also present the user feedback and participation.

**Discussion:**

PPIs are central to cellular systems. Yet less than 10% of estimated PPIs are known today. Thus, the computationally predicted PPIs which are deemed accurate, can accelerate advancement of schizophrenia biology research. The time is ripe to benefit from computer science and information technologies methods for not only discovering aspects of computational biology but to create new mechanisms to promote online collaboration to achieve big things as a summation of nanocontributions. The knowledge potential generated through this system would aid various principal investigators in well established (but ill funded) research labs by giving them access to bioinformaticians and biologists around the world who are eager be recognized for their contributions. We present here, not merely a website but a novel approach to promote collaborative research between people with heterogeneous skills and commitments by benefiting from the untapped talent of researchers around the world.

